# Methods for confidence interval estimation of a ratio parameter with application to location quotients

**DOI:** 10.1186/1471-2288-5-32

**Published:** 2005-10-12

**Authors:** Joseph Beyene, Rahim Moineddin

**Affiliations:** 1 Department of Public Health Science, University of Toronto, Toronto, Ontario, Canada; 2 The Research Institute, Hospital for Sick Children, Toronto, Ontario, Canada; 3 Department of Family and Community Medicine, Research Program, University of Toronto, Toronto, Ontario, Canada

## Abstract

**Background:**

The location quotient (LQ) ratio, a measure designed to quantify and benchmark the degree of relative concentration of an activity in the analysis of area localization, has received considerable attention in the geographic and economics literature. This index can also naturally be applied in the context of population health to quantify and compare health outcomes across spatial domains. However, one commonly observed limitation of LQ is its widespread use as only a point estimate without an accompanying confidence interval.

**Methods:**

In this paper we present statistical methods that can be used to construct confidence intervals for location quotients. The delta and Fieller's methods are generic approaches for a ratio parameter and the generalized linear modelling framework is a useful re-parameterization particularly helpful for generating profile-likelihood based confidence intervals for the location quotient. A simulation experiment is carried out to assess the performance of each of the analytic approaches and a health utilization data set is used for illustration.

**Results:**

Both the simulation results as well as the findings from the empirical data show that the different analytical methods produce very similar confidence limits for location quotients. When incidence of outcome is not rare and sample sizes are large, the confidence limits are almost indistinguishable. The confidence limits from the generalized linear model approach might be preferable in small sample situations.

**Conclusion:**

LQ is a useful measure which allows quantification and comparison of health and other outcomes across defined geographical regions. It is a very simple index to compute and has a straightforward interpretation. Reporting this estimate with appropriate confidence limits using methods presented in this paper will make the measure particularly attractive for policy and decision makers.

## Background

Effects in comparative analysis are commonly expressed as ratios. One such example is the Location Quotient (LQ), a ratio statistic widely used by geographers, economists and regional planners to measure the degree of relative concentration of an activity on a map [1,2]. The LQ, which is sometimes referred to as concentration ratio, allows the comparison of an area's share of a specific activity with the share of a base aggregate. Furthermore, LQ can produce a rough benchmark in the analysis of localization in an area [3].

In general, statistical inference is more complicated for a ratio of parameters than measures that are expressed as linear combinations. In epidemiological studies, for example, association of risk factors with occurrence of disease in a given study population can be quantified using absolute measures such as the risk difference or by applying relative measures such as the relative risk or odds ratio [4]. Both the relative risk and the odds ratio require more caution from an inferential point of view than the simple risk difference. One of the difficulties in dealing with ratios arises in computing variance estimators.

Despite its popularity as a relative measure, the location quotient is often interpreted and reported primarily as a point estimate without an accompanying measure of precision. However, statistical reasoning and any inferential conclusion one may draw from a sample statistics should reflect uncertainty inherent in the estimation procedure, and appropriate methods that allow proper interpretation of study findings should be used. One way of proper analysis is to construct confidence limits around the sample estimates.

The objective of this paper is to present a number of alternative approaches that can be used to construct confidence limits for measures involving ratios quantities in general and the location quotient in particular.

## Methods

A location quotient is a way of measuring the relative contribution of one specific area to the whole for a given outcome. Let *x*_*i *_and *n*_*i *_denote the outcome and population size of the i^th ^area, respectively. Similarly, let *x *= ∑*x*_*i *_and *n *= ∑*n*_*i *_be the outcome and population size of the whole, respectively. The location quotient for the i^th ^area is defined as



Depending upon the health outcome under study, the random variables in equation (1) may have different scales of measurements including continuous, binary or counts.

The interpretation of the LQ is as follows: (1) *LQ*_*i *_= 1 which indicates that the outcome in the specific region is at the same level as the aggregate, (2) *LQ*_*i *_> 1 indicating that the specific region is at a level greater than expected, and (3) *LQ*_*i *_< 1 which would indicate that the regional measure is at a level that is less than expected.

To fix notations, suppose interest lies in making inference about a ratio parameter . In this paper, our focus is on confidence interval estimation of θ. Let  be an estimate of θ, where the mean parameters for the estimates are given by *E*() = α and *E*() = β, respectively. Furthermore, let the estimated variance-covariance matrix of the estimators (, ) be given by



where *V*_11 _and *V*_22 _represent the variance of  and , respectively, and *V*_12 _= *V*_21_denote the covariance between  and .

For the location quotient described in equation (1), , .

It can easily be shown that



where the parameter *p*_*i *_denotes the true incidence rate in the i^th ^area [5].

Using the notation introduced above, we now describe three analytical and computational approaches that can be used to construct confidence intervals for ratio parameters, namely: (1) Delta method (2) Fieller's method and (3) profile-likelihood based interval on generalized linear model (GLM) technique.

### The delta method

The delta method is a classic technique in statistics that is based on a truncated Taylor series expansion [6]. According to the delta method, the variance of  is estimated by



For sufficiently large sample size, one may assume that  has a Gaussian distribution with mean θ and variance σ^2 ^from which a (1 - α)% delta-method based confidence interval can be obtained as



where *z*_α/2 _is the (1 - α/2)% quintile of the standard normal distribution (for instance, for a 95% confidence interval α = 0.05 and *z*_α/2 _= 1.96) and  is the square-root of the expression in equation (3).

### The Fieller method

Fieller [7] introduced a novel way of expressing ratios as linear combination of random variables which made computation of confidence intervals of ratios relatively simple.

The justification for Fieller's method proceeds as follows. Suppose  and  have a bivariate normal distribution with mean vector (α, β)' and variance-covariance matrix as given in equation (2). If we let , then it follows that α + θβ = 0. Now consider the linear combination  + θ = 0. It is a well known fact of mathematical statistics that the distribution of a linear combination of normally distributed random variables is itself normal. In particular, it can be shown that



where σ^2 ^= (*V*_11 _+ 2θ*V*_12 _+ θ^2^*V*_22_). This result implies that  is a standard normal random variable and its square is a chi-squared variable with 1 degree of freedom, χ_1_^2^.

A (1 - α)% Fieller confidence interval is then obtained by finding the set of θ values satisfying the inequality



Equation (6) is a quadratic function in the parameter of interest θ and solving for θ leads to the confidence limits



where .

Both the delta method and Fieller's approach are quite generic and have been used in a wide range of applications [8-10]. The implementation of these two approaches (using equations (4) and (7) respectively neither require sophisticated programming nor specialized software.

### Generalized linear modelling

A model that is widely applicable in a number of different distributional scenarios is generalized linear model (GLM) [11]. Among others, the normal, binomial and Poisson distributions are included in this rich family of models.

We consider a situation where we have *k *regions and need to estimate *k *location quotients along with the corresponding confidence limits. We formulate the generalized linear framework by re-expressing equation (1) as

log(*p*_*i*_) = log(*x*/*n*) + β_1_*I*_1 _+ … + β_*k*_*I*_*k*_,     (8)

where *p*_*i *_is estimated by  and the link function relating the outcome to the independent variables is a logarithmic transformation. The indicator variables *I*_*j*_, *j *= 1,…,*k *take on the value 1 if the region is *j *and 0 otherwise.

The estimated regression coefficients  in the above model provide a point estimate of the location quotient in each area in a logarithmic scale. Exponentiating these estimates give the location quotients in their natural scale. For example, if the region of interest is *region 1*, then the indicator variable *I*_1 _will take the value 1 and the remaining indicator variables will be zero. In this case, equation (8) becomes log(*x*_1_/*n*_1_) = log(*x*/*n*) + β_1_, and a simple re-arrangement shows that the estimated regression coefficient is expressed as . Exponentiating this result leads to , which is the location quotient for *region-1*.

There are a number of attractive features with this formulation. Firstly, the model can be fitted using standard statistical software such as using the GENMOD procedure in the SAS statistical package [12]. The resulting estimators are maximum likelihood estimators that are well known to have desirable optimality properties. In fact the reason we were able to take the anti-logarithm (using exponential) to get back to the natural scale for the location quotients from the logarithmic scale was due to the invariance property of the maximum likelihood estimates. The invariance property ensures that, if  is the maximum likelihood estimator of θ, then *g*() is a maximum likelihood estimator of *g*(θ).

Secondly, confidence intervals for model parameters are by products of the modelling procedure. One of the intervals that can be extracted from fitting the generalized linear model is the profile-likelihood based interval. This approach is an iterative procedure which in general gives more accurate confidence limits, especially for small sample sizes [13]. In addition, significance levels are generated automatically that can be used along side the confidence intervals in order to test whether or not the LQ for a given region is significantly different from the null value of one (*H*_0_: *LQ *= 1) or to make comparisons across different regions.

Thirdly, as mentioned earlier, the GLM family encompasses a large number of commonly used statistical distributions. Thus one can use this framework for modelling indices that are based on health outcome measurements with different scales including continuous scale and categorical outcomes.

## Results

### Simulation results

A simulation study was carried out to investigate the performance of the methods for calculating confidence limits for the location quotient. Three areas with varying population sizes and incidence rates were considered. Table 1 summarizes confidence limits for the resulting three location quotients based on 1000 simulated data sets within each configuration. The average 2.5%-ile and 97.5%-ile values, shown in Table 1 along the rows designated by method "S", were used as "benchmarks" to compare the performance of the delta (D), Fieller (F), and profile-likelihood (P) methods.

**Table 1:** Comparison of 95% confidence intervals for three location quotients (LQ^1^, LQ^2^, LQ^3^) using three methods (D = delta, F = Fieller, P = Profile-likelihood). Varying incidence rates (*p*_1_, *p*_2_, *p*_3_) were used along with 3 sets of population size configurations (a) *n*_1 _= 50, *n*_2 _= 80, *n*_3 _= 60 (b) *n*_1 _= 500, *n*_2 _= 900, *n*_3 _= 100 (c) *n*_1 _= 2000, *n*_2 _= 2500, *n*_3 _= 1500. A total of 1000 simulated data sets were used to generate "benchmark" limits (designated as "S" under method)

	*p*_1_	*p*_2_	*p*_3_	Method						
(a)	0.25	0.3	0.2	S	0.5903	1.3959	0.8787	1.4844	0.4332	1.1237
				D	0.5789	1.3905	0.8860	1.4679	0.4380	1.1155
				F	0.5664	1.4043	0.8815	1.4824	0.4239	1.1234
				P	0.5715	1.4995	0.8109	1.5950	0.4375	1.2183
	0.2	0.4	0.7	S	0.2249	0.6926	0.7125	1.0744	1.3548	1.8404
				D	0.2235	0.6776	0.7168	1.0909	1.3309	1.8413
				F	0.2156	0.6758	0.7126	1.0918	1.3464	1.8651
				P	0.2409	0.7287	0.6719	1.1505	1.3096	1.8251
	0.9	0.1	0.5	S	1.8387	2.3249	0.0956	0.3765	0.9383	1.3492
				D	1.7335	2.3942	0.0901	0.3640	0.9040	1.3841
				F	1.7718	2.4478	0.0846	0.3624	0.9044	1.3910
				P	1.8297	2.2054	0.1088	0.4035	0.8607	1.4280
	0.02	0.01	0.1	S	0.0000	1.6889	0.0000	1.0037	1.2667	3.1667
				D	-0.1536	1.1816	-0.0924	0.6015	1.4479	3.3499
				F	-0.5404	1.5539	-0.3073	0.7883	1.0254	4.2516
				P	0.3015	1.0973	0.1272	0.6214	1.9683	3.1071

(b)	0.25	0.3	0.2	S	0.7924	1.0245	1.0156	1.1439	0.4483	1.0191
				D	0.7917	1.0197	1.0167	1.1489	0.4517	1.0012
				F	0.7911	1.0199	1.0167	1.1493	0.4504	1.0017
				P	0.7731	1.0476	0.9765	1.1932	0.4736	1.0343
	0.2	0.4	0.7	S	0.4766	0.6536	1.0807	1.1854	1.7154	2.2426
				D	0.4789	0.6537	1.0755	1.1865	1.7326	2.2467
				F	0.4781	0.6533	1.0757	1.1870	1.7370	2.2525
				P	0.4715	0.6698	1.0411	1.2225	1.7240	2.2265
	0.9	0.1	0.5	S	2.2140	2.3684	0.2129	0.2938	1.0390	1.5164
				D	2.1557	2.4273	0.2084	0.2971	1.0277	1.5079
				F	2.1629	2.4354	0.2078	0.2967	1.0281	1.5093
				P	2.2196	2.3530	0.2060	0.3053	1.022	1.5138
	0.02	0.01	0.1	S	0.5071	1.5517	0.2381	0.8333	2.5862	7.9550
				D	0.5212	1.5436	0.2396	0.7928	2.6717	7.7130
				F	0.4819	1.5835	0.2174	0.8136	2.5448	7.9803
				P	0.5205	1.8078	0.2494	0.9322	2.6765	8.8046

(c)	0.25	0.3	0.2	S	0.9113	1.0322	1.1066	1.2119	0.7077	0.8480
				D	0.9089	1.0296	1.1088	1.2115	0.7043	0.8439
				F	0.9088	1.0296	1.1089	1.2117	0.7041	0.8438
				P	0.8970	1.0440	1.0914	1.2305	0.6977	0.8544
	0.2	0.4	0.7	S	0.4518	0.5288	0.9424	1.0115	1.6636	1.7688
				D	0.4519	0.5285	0.9427	1.0147	1.6588	1.7717
				F	0.4518	0.5283	0.9427	1.0147	1.6595	1.7726
				P	0.4482	0.5341	0.9319	1.026	1.6577	1.7712
	0.9	0.1	0.5	S	1.8962	1.9663	0.1923	0.2365	1.0271	1.1150
				D	1.8797	1.9797	0.1903	0.2374	1.0237	1.1176
				F	1.8808	1.9808	0.1902	0.2373	1.0237	1.1177
				P	1.9004	1.9567	0.1896	0.2399	1.0165	1.1249
	0.02	0.01	0.1	S	0.4000	0.7184	0.1798	0.3847	2.5421	3.0220
				D	0.4020	0.7094	0.1776	0.3813	2.5418	3.0449
				F	0.4005	0.7106	0.1766	0.3821	2.5428	3.0504
				P	0.4018	0.7441	0.1844	0.4026	2.3876	3.2374

Panel (a) in Table 1 shows results when area population sizes are relatively *small *with *n*_1 _= 50, *n*_2 _= 80, *n*_3 _= 60, For this scenario, the results from the different approaches can differ, specially when the incidence rates are also small. For instance, when the three incidence rates are set to *p*_1 _= 0.02, *p*_2 _= 0.01, and *p*_3 _= 0.10, both the delta and Fieller intervals resulted in negative lower limits, which would obviously be inappropriate for location quotients. In such cases, the profile-likelihood method may be preferable. On the other hand, we observe a remarkable agreement among the different methods when population sizes are relatively *large *with *n*_1 _= 2000, *n*_2 _= 2500, *n*_3 _= 1500. In this case, the accuracy of the results is quite good even when the incidence rates are small, i.e., the last 4 rows of Table 1. Panel (b) provides results for *moderate *population sizes with *n*_1 _= 500, *n*_2 _= 900, *n*_3 _= 100. Overall, the three methods lead to quite similar confidence intervals in this situation, with the delta method and Fieller's intervals being more close to each other.

### Application to health utilization data

In this section, the different methods of estimating confidence interval for the location quotient are illustrated using a health utilization data set from Ontario, Canada. Data were extracted from the Ontario Health Insurance Plan (OHIP) database for all ambulatory specialist visits due to rheumatoid arthritis in the fiscal year 1996. The visits were assigned to census divisions based on where the patient was registered and not where services were received. Forty four censuses divisions were used for analysis. For each county, the LQ is defined as the ratio of two proportions with the numerator representing the number of visits to rheumatologists in the census division divided by the total number of specialist visits in the census division, and the denominator defined as the number of visits to rheumatologists for the province divided by the total number of specialist visits in the province.

For a given county, a location quotient of less than one indicates that the utilization of health care services is under represented compared to the provincial utilization rate. On the other hand, a location quotient of greater than one suggests that health care services utilization is greater than expected. A location quotient of one indicates lack of under or over concentration of utilization in the census division.

Table 2 shows the data, along with the estimated location quotients and confidence intervals. The intervals based on the delta and Fieller's methods were identical to three decimal places. Therefore only the Fieller's lower and upper limits are shown in Table 2 and compared with the intervals based on the profile-likelihood results in the generalized linear models.

**Table 2:** Location quotients for health utilization data, with 95% lower and upper confidence limits using (1) Fieller's and (2) Profile-likelihood methods (see text for details about the data and methods)

County	*n*_*i*_	*x*_*i*_	*LQ*_*i*_	Fieller Lower	Fieller Upper	Profile Lower	Profile Upper
1	975	365	0.779	0.716	0.842	0.717	0.843
2	370	115	0.647	0.549	0.745	0.552	0.747
3	275	155	1.173	1.051	1.295	1.050	1.293
4	635	170	0.557	0.486	0.629	0.487	0.631
5	2405	1835	1.588	1.552	1.623	1.552	1.622
6	655	175	0.556	0.486	0.626	0.487	0.628
7	730	335	0.955	0.880	1.030	0.880	1.030
8	115	60	1.086	0.896	1.276	0.896	1.273
9	3205	1545	1.003	0.968	1.038	0.967	1.039
10	500	220	0.916	0.825	1.006	0.826	1.007
11	365	125	0.713	0.611	0.814	0.614	0.816
12	915	435	0.989	0.922	1.056	0.922	1.057
13	4920	225	0.095	0.083	0.107	0.084	0.108
14	500	315	1.311	1.223	1.399	1.222	1.397
15	525	215	0.852	0.765	0.939	0.766	0.940
16	20770	11035	1.106	1.093	1.118	1.091	1.120
17	3025	1595	1.097	1.061	1.134	1.060	1.134
18	350	155	0.922	0.813	1.030	0.814	1.030
19	4500	1675	0.775	0.746	0.803	0.745	0.804
20	610	460	1.569	1.498	1.640	1.496	1.638
21	3915	2780	1.478	1.448	1.507	1.448	1.507
22	10550	6710	1.323	1.305	1.342	1.304	1.343
23	720	250	0.723	0.650	0.795	0.651	0.796
24	6080	3130	1.071	1.046	1.096	1.045	1.097
25	350	140	0.832	0.726	0.939	0.727	0.940
26	1840	1140	1.289	1.244	1.335	1.243	1.335
27	685	500	1.519	1.450	1.588	1.448	1.586
28	920	405	0.916	0.850	0.982	0.850	0.983
29	2585	1395	1.123	1.084	1.162	1.083	1.163
30	1020	345	0.704	0.644	0.764	0.644	0.765
31	770	470	1.270	1.199	1.342	1.198	1.341
32	4240	1515	0.744	0.714	0.773	0.714	0.774
33	1580	205	0.270	0.236	0.304	0.237	0.306
34	5505	2475	0.936	0.909	0.962	0.908	0.963
35	3665	2420	1.374	1.343	1.405	1.342	1.406
36	1145	310	0.563	0.510	0.617	0.511	0.618
37	485	100	0.429	0.354	0.504	0.357	0.507
38	400	210	1.092	0.991	1.194	0.991	1.194
39	170	50	0.612	0.470	0.754	0.477	0.760
40	610	115	0.392	0.328	0.457	0.330	0.460
41	380	115	0.630	0.534	0.726	0.537	0.728
42	2695	865	0.668	0.632	0.704	0.632	0.705
43	1865	540	0.603	0.560	0.645	0.560	0.646
44	165	30	0.378	0.256	0.501	0.267	0.511

Total	98685	47425					

There is a remarkable agreement in the confidence intervals from both the Fieller and profile-likelihood approaches due to the fact that the denominator of the ratio is estimated with sufficiently high precision, which would be the case for applications with large sample sizes. Small relative differences are observed when sample sizes are small, as in counties 39 and 44 in Table 2.

The LQ was significantly greater than 1, which is the entire confidence interval falls above 1, for 32% (14/44) of the census divisions, indicating significantly higher utilization of health care services for rheumatoid arthritis than the provincial rate. Similarly, 52% (23/44) of the census divisions showed a significantly lower utilization rate. The remaining 16% experienced a utilization rate compatible with the provincial rate.

## Discussion

The location quotient (LQ) is one of several spatial measures that is widely used to examine spatial variation of area characteristics [14]. However, a frequently occurring 'gap' is the widespread use of LQ as a point estimate without an accompanying confidence interval. In this paper, we have demonstrated that confidence intervals for ratio parameters in general and location quotients in particular can be obtained using a number of complimentary approaches. Three techniques – the delta method, Fieller's interval, and profile-likelihood based interval from a generalized linear model – are presented and illustrated. We also demonstrated that if the denominator of the ratio is estimated with sufficiently high precision, then the methods introduced in this paper will produce very similar confidence intervals. The normal approximation to the binomial is used for the variance estimate for the calculation by the delta and Fieller methods. Hence it is not surprising that these methods do not perform well for small sample sizes and extreme proportions.

The techniques we described are generic and can be applied to a wide range of settings where ratio parameters are in use. The generalized linear model (GLM) approach is particularly appealing since the parameters of interest (in our application the location quotients) are estimated directly along with confidence intervals and significance levels. These considerations can be important in practical applications where there are several parameters to be estimated. For instance, for the health utilization data set we analyzed in this paper, 44 location quotients and their confidence intervals had to be generated and the modelling approach was the preferred method over the delta and Fieller's techniques.

## Competing interests

The author(s) declare that they have no competing interests.

## Authors' contributions

JB initiated, designed, and drafted the study. Both JB and RM participated in simulating, analyzing, and discussing the results and in writing the paper. All authors read and approved the final manuscript.

## Pre-publication history

The pre-publication history for this paper can be accessed here:


